# Vanishing hypercalciuric kidney stones after treating underlying acromegaly

**DOI:** 10.1530/EDM-13-0001

**Published:** 2013-07-01

**Authors:** Eline van der Valk, Tom Tobe, Aline Stades, Alex Muller

**Affiliations:** Diakonessenhuis Utrecht, Department of Internal MedicinePostbus 80250, 3508 TG, UtrechtThe Netherlands; 1 UMC Utrecht, Department of EndocrinologyPostbus 85500, 3508 GA, UtrechtThe Netherlands

## Abstract

A 53-year-old male presented with recurrent calcium oxalate kidney stones as a first sign of underlying acromegaly, which vanished when his acromegaly was controlled. The exact mechanism behind hypercalciuria and urolithiasis in acromegaly is not yet clear. By discussing this case, a short overview of the pathophysiology of hypercalciuria in acromegaly and practical insights are given.

**Learning points:**

- Hypercalciuria is a common finding in acromegaly.
- There are only few reports describing hypercalciuric kidney stones in acromegaly.
- We assume that in acromegaly there is a primary role of IGF1-mediated, PTH-independent increase in calcitriol synthesis resulting in hypercalciuric kidney stones.

## Background

Acromegaly is a multisystem disease with rheumatological, cardiovascular, respiratory and metabolic consequences (1). A frequent metabolic consequence is hypercalciuria (2) (3). Although several studies have been published on this subject, the exact mechanism is not yet clear. Moreover, nephrolithiasis with calcium-containing stones occurs more frequently in patients with acromegaly, presumably as a consequence of this hypercalciuria (2) (4) (5) (6) (7).

Here, we report a case of a patient in whom calcium oxalate kidney stones led to an underlying diagnosis of acromegaly and in whom treatment of acromegaly resulted in the disappearance of these kidney stones.

## Case presentation

A 53-year-old male with a medical history of hypertension, type 2 diabetes mellitus, osteoarthritis and obstructive sleep apnoea was referred because of recurrent kidney stones. For the past 18 months, he had excreted approximately one renal stone each month. Other symptoms included dental problems, an enlarged tongue, thickened skin, increased perspiration and enlarged feet and hands that had developed over the past few years. Besides his acromegalic features, the physical examination was normal. Analysis of the stones revealed that they were made up of 100% calcium oxalate. Additional testing showed normal concentrations of calcium, phosphate, parathyroid hormone (PTH) and vitamin D and normal urinary phosphate excretion (Table 1). There was no evidence of sarcoidosis, as an X-ray showed no abnormalities, and angiotensin-converting enzyme levels were normal.

**Table 1:** Biochemical and hormonal parameters in active and controlled acromegaly

**Biochemical and hormonal parameters**	**Active acromegaly**	**Controlled acromegaly**	**Reference value**
Plasma albumin (g/l)	42.5	43	35–55
Creatinine (μmol/l)	95	123	50–120
Calculated clearance (ml/min)	129	96	>60
Plasma Ca (mmol/l)	2.43	2.28	2.10–2.55
Albumin-corrected plasma calcium (mmol/l)	2.5	2.2	2.10–2.55
Plasma Mg (mmol/l)	0.72	0.79	0.65–1.05
Plasma PO_4_ (mmol/l)	1.45	1.22	0.75–1.40
Serum IGF1 (ng/ml)	762	161	50–175
Plasma PTH (pmol/l)	2.9	7.7	2.0–7.0
Plasma 25-OH-vitamin D	72.6	53.7	17.7–113.3
Plasma 1,25(OH)_2_-vitamin D	Not available	85.7	47.0–130.3
24-h U volume (l/24 h)	2000	1950	
24-h U creatinine excretion (mmol/24 h)	18.5	16.8	10–42
24-h U calcium excretion (mmol/24 h)	6.0	2.0	2.5–5.0
24-h U PO_4_ excretion (mmol/24 h)	29.8	45.8	10.0–40.0
24-h oxalate excretion (μmol/24 h)	672	522	<444
24-h citrate excretion (mmol/24 h)	4.52	1.28	1.0–3.5

## Investigation

**Figure 1:**
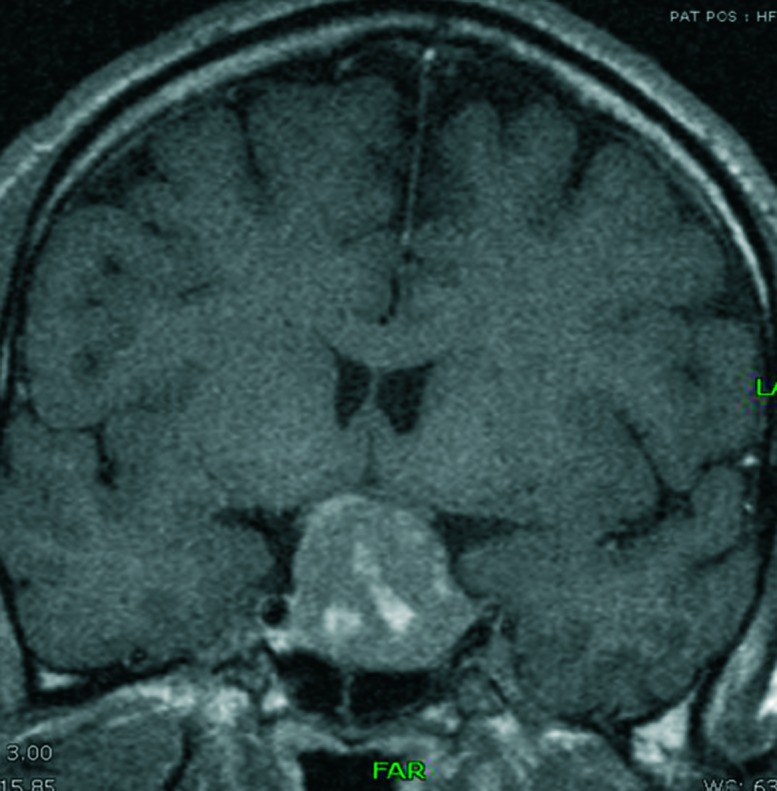
MRI scan showing a pituitary macro-adenoma with supra- and parasellar extension.

Considering the clinical presentation, acromegaly was suspected and the IGF1 level was found to be significantly increased. MRI of the brain revealed a pituitary macro-adenoma with supra- and parasellar extension (Fig. 1). There was secondary hypogonadism, but pituitary–adrenal and pituitary–thyroid axes were unaffected.

## Treatment

The patient was referred for neurosurgery and prescribed a long-acting somatostatin analogue.

## Outcome and follow-up

After initiation of treatment with a somatostatin analogue, excessive perspiration disappeared, the apnoeas were less frequent and the frequency of hypoglycaemia increased. Hypertension was better controlled. Nine months after diagnosis, the patient underwent endoscopic transnasal transsphenoidal selective adenomectomy without complications. After the operation and after cessation of the somatostatin analogue treatment, his IGF1 level was normal and he became normoglycaemic without medication. Calcium excretion normalised, and no kidney stones have been passed since then.

**Figure 2:**
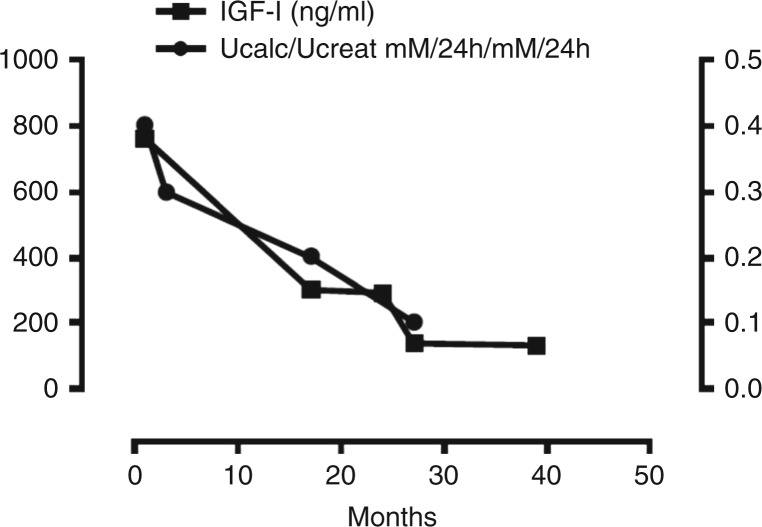
IGF1 and Ucalc/Ucreat in time.

Interestingly, a direct correlation between calcium excretion and IGF1 is seen (Fig. 2). This corresponds to the clinical presentation, as he had no more kidney stones after initiation of the treatment.

## Discussion

In most studies, it is assumed that increased calcitriol synthesis is at least partially responsible for hypercalciuria in acromegaly (8) (9) (10) (11). From rat studies, it is known that acromegaly promotes calcitriol synthesis, either through the effect of GH or through the effect of IGF1 or both (8) (10). Currently, it is assumed that IGF1 directly promotes renal α-hydroxylase activity, leading to increased calcitriol levels and thus enhanced intestinal dietary calcium absorption with subsequent hypercalciuria (12) (13) (14). However, in a recent study, it has been shown that the calcitriol-mediated effect of acromegaly also involves increased distal tubular calcium reabsorption (3). So, in acromegaly, there is absorbtive hypercalciuria, which is only partly counteracted by increased distal tubular calcium reabsorbtion, leading to a net effect of hypercalciuria. The data presented in Fig. 2 are in full accordance with a primary role for IGF1 in the mechanism by which acromegaly leads to hypercalciuria.

PTH most likely has no causal role in the development of hypercalciuria in acromegaly. Most studies have reported low-normal PTH levels in uncontrolled acromegaly, which increase after adequate treatment (3) (15) (16). Others have reported normal PTH levels that are not specified (11) (17). One study found high PTH levels in acromegalic patients that increase slightly after treatment, which is also suggestive of no causal role for PTH (9).

It is evident that hypercalciuria occurs in acromegaly, and therefore, one would expect a high incidence of nephrolithiasis in acromegaly. There are, however, surprisingly few case reports of urolithiasis in acromegaly. Auriemma *et al*. (2) reviewed the mechanism of kidney stones with respect to acromegaly. They found hypercalciuria, hyperoxaluria and hypercitraturia and explained the low incidence of urolithiasis by the presence of hypercitraturia, which prevents the formation of urolithiasis (18) (19) (20). In the patient described here, urinary citrate concentration also decreased after controlling his acromegaly, which supports the mechanism observed by Auriemma *et al*. However, in our case, renal colic disappeared completely in parallel with a lowering of citraturia, suggesting that hypercalciuria was the driving force of renal stone formation in our patient.

Recently, it has been suggested that bone is an active regulator of energy and glucose metabolism (21). Iba *et al*. (22) first described a correlation between metabolic syndrome and hypercalciuria in animals. Liborio *et al*. (23) have recently investigated the correlation between insulin resistance and hypercalciuria in acromegaly. Insulin resistance appears to be associated with hypercalciuria, but it is still unclear whether this association is causal, as active acromegaly is associated with both insulin resistance and hypercalciuria. Perhaps both hypercalciuria and insulin resistance are merely symptoms of the same underlying active disease. The patient we describe here became normoglycemic without medication after control of his acromegaly, and therefore, we cannot draw a clear conclusion regarding the possible causal role of insulin resistance in the development of hypercalciuria in acromegaly. In our opinion, there are no strong arguments to suggest a role for insulin resistance as a cause for hypercalciuria in acromegaly.

In conclusion, we describe a patient in whom the presence of hypercalciuric kidney stones led to the diagnosis of acromegaly and in whom the kidney stones vanished after treatment of his acromegaly. This case is in full accordance with a primary role of IGF1-mediated, PTH-independent increase in calcitriol synthesis resulting in hypercalciuric kidney stones.

## Patient consent

Written informed consent was obtained from the patient for publication of this case report. 

## Author contributions

E van der Valk was responsible for case description, literature review and writing. T Tobe was the patient's physician and was responsible for editing. A Stades was responsible for literature review and editing. A Muller was the physician of the patient and was responsible for literature review, writing and editing.
